# Improving data quality and supervision of antiretroviral therapy sites in Malawi: an application of Lot Quality Assurance Sampling

**DOI:** 10.1186/1472-6963-12-196

**Published:** 2012-07-09

**Authors:** Bethany L Hedt-Gauthier, Lyson Tenthani, Shira Mitchell, Frank M Chimbwandira, Simon Makombe, Zengani Chirwa, Erik J Schouten, Marcello Pagano, Andreas Jahn

**Affiliations:** 1 Department of Biostatistics, Harvard School of Public Health, Boston, MA, USA; 2 Department of Global Health and Social Medicine, Harvard Medical School, Boston, MA, USA; 3 Department for HIV and AIDS, Ministry of Health, Lilongwe, Malawi; 4 I-TECH, Malawi and University of Washington, Seattle, WA, USA; 5 Management Sciences for Health, Lilongwe, Malawi

**Keywords:** HIV treatment programs, Malawi, Africa, Supervision, Data quality

## Abstract

**Background:**

High quality program data is critical for managing, monitoring, and evaluating national HIV treatment programs. By 2009, the Malawi Ministry of Health had initiated more than 270,000 patients on HIV treatment at 377 sites. Quarterly supervision of these antiretroviral therapy (ART) sites ensures high quality care, but the time currently dedicated to exhaustive record review and data cleaning detracts from other critical components. The exhaustive record review is unlikely to be sustainable long term because of the resources required and increasing number of patients on ART. This study quantifies the current levels of data quality and evaluates Lot Quality Assurance Sampling (LQAS) as a tool to prioritize sites with low data quality, thus lowering costs while maintaining sufficient quality for program monitoring and patient care.

**Methods:**

In January 2010, a study team joined supervision teams at 19 sites purposely selected to reflect the variety of ART sites. During the exhaustive data review, the time allocated to data cleaning and data discrepancies were documented. The team then randomly sampled 76 records from each site, recording secondary outcomes and the time required for sampling.

**Results:**

At the 19 sites, only 1.2% of records had discrepancies in patient outcomes and 0.4% in treatment regimen. However, data cleaning took 28.5 hours in total, suggesting that data cleaning for all 377 ART sites would require over 350 supervision-hours quarterly. The LQAS tool accurately identified the sites with the low data quality, reduced the time for data cleaning by 70%, and allowed for reporting on secondary outcomes.

**Conclusions:**

Most sites maintained high quality records. In spite of this, data cleaning required significant amounts of time with little effect on program estimates of patient outcomes. LQAS conserves resources while maintaining sufficient data quality for program assessment and management to allow for quality patient care.

## Background

In 2004, the Malawi Ministry of Health (MoH) began the rapid expansion of the national HIV treatment program, initiating 271,105 and retaining 198,846 patients on antiretroviral therapy (ART) at 377 sites (279 static and 98 mobile/outreach) by October 2009 [1]. The successful scale-up of the Malawi ART program was made possible through a public health approach [2]. ART eligibility is determined by WHO clinical staging or a low CD4 count. In 2009, these criteria were Stage III or IV disease or CD4 < 250 cells/mm^3^ (CD4 < 350 cells/mm^3^ for pregnant women). One standard, fixed-dose first-line regimen (stavudine, lamivudine, nevirapine in 2009) is used for >90% of patients and standard alternative regimens are used for patients who develop side effects to the first-line regimen [3]. Experienced sites (generally district hospitals that have been offering ART for at least three years) also provide standard second-line regimens for eligible patients. Patients visit ART clinics monthly until stable on therapy (usually after six to ten months). Two- or three-monthly appointments are scheduled thereafter.

Two critical components of the national ART program are 1) a standardized system for registering and monitoring patients, including individual patient treatment cards with registration and follow-up data and a clinic register summarizing critical data for each patient [3] and 2) quarterly supervision at all ART sites in Malawi [4]. Every quarter, ART clinic personnel perform a standardized patient cohort analysis that includes aggregation of case-finding details of patients registered during the previous quarter and since ART was first begun, as well as treatment outcomes for the cumulative cohort. The latter analysis requires a review of all treatment cards in order to update the clinic register and then a tally-score on current patient outcomes. The primary outcome is patient status, namely, alive on ART, died, stopped ART (with clinician’s knowledge), transferred to another ART clinic, or lost to follow-up. Secondary outcomes for patients alive on ART include 1) ART regimen, 2) drug adherence, 3) ART side effects, and 4) current tuberculosis status. A rolling cohort survival analysis is also performed by counting primary outcomes of patients who registered during specified previous quarters [2].

During quarterly supervision, teams of at least two individuals (including a representative from the MoH national or regional office along with a MoH clinical officer or nurse) visit each site for up to a full day. Additional representatives from the health sector, including other MoH staff or non-governmental organization partners, join teams on occasion. Currently, quarterly supervision requires ten teams to be in the field for two to three weeks each at a cost of approximately $60,000 per quarter (excluding staff salaries). The quarterly supervision activity is coordinated by the monitoring and evaluation (M&E) team at the Department for HIV and AIDS in the MoH and funded through the U.S. President's Emergency Plan for AIDS Relief (PEPFAR) and the Global Fund to Fight AIDS, Tuberculosis and Malaria. These visits provide an opportunity to identify any problems at the sites, to discuss difficult cases with medical staff, and to monitor drug stocks, thus improving the overall quality of care [5].

The supervision teams also review the patient cohort analyses produced by the ART staff and clean patient data and aggregate quarterly and cumulative reports on 44 data elements. These data are used for drug forecasting, monitoring overall program performance, identifying gaps in care, and as early warning indicators for HIV drug resistance [6-18], and therefore maintaining high quality data is of utmost importance. Like other national treatment programs, Malawi has strived since its inception to provide complete, accurate and up-to-date data. Common errors include incomplete registers, failure to update follow-up outcomes, duplicate entries, and incorrect/missing records [5]. In 2008, Makombe et al reported that unchecked site-produced reports resulted in a 12% undercount of patients on ART, affecting the programs ability to precisely forecast future drug needs [19].

Currently every patient treatment card is reviewed and compared against the entries in the register during supervision visits to ensure up-to-date and accurate data in the register prior to data extraction. Inconsistencies or incomplete data are updated as appropriate. This process can take many hours at each visit, competing with other important aspects of supervision and mentoring. Further, this *full* audit of patient records by the supervision team may not yield any changes, especially at sites where experienced clinic staff have correctly updated treatment cards and clinic registers in preparation for the supervision visit.

While the quality of recording for certain key elements (such as outcome status or regimen) seem to have improved with repetition of this intensive data cleaning process, there remain significant challenges in quality of secondary elements, such as pill counts and side effects. The goal of this study was to analyze the types and frequency of data errors currently observed in the national HIV treatment program and to evaluate their impact on management and monitoring. Based on these findings we evaluated the performance of a lot quality assurance sampling (LQAS) algorithm to prioritize ART sites with poor data quality for a full review of their records before aggregation of quarterly reports. We then estimated the potential amount of time saved by omitting an exhaustive review of all treatment cards at sites that pass the LQAS assessment. Further, we estimated secondary outcomes from treatment cards during the sampling process.

## Methods

### Site Visits

From 11 to 22 January 2010, a study team joined supervision teams for the 4^th^ Quarter (Q4) 2009 supervision. Twenty-three sites were chosen. Selection was not random, but purposely sampled to achieve diverse representations in terms of geography, size, length of providing treatment, and public/private sectors. As per MoH standard, the clinics were informed of the visit by the supervision team but were not aware that the team would be accompanied by a study team. Although the study team visited all 23 clinics; four were excluded because they either had not initiated any patients on treatment or the patient treatment card filing system had not yet been created. At each site, the study team recorded every treatment card/register discrepancy identified by the supervision team including the type of discrepancy, the information recorded in the treatment card and register, and the correct (updated) information. The study team randomly sampled from the list of all patients enrolled at the site, found the corresponding treatment cards, and recorded the time required for sampling and selected secondary outcomes of patients still alive and on treatment (side effects and treatment adherence; the latter was defined by MoH as eight or fewer pills remaining since last visit). If a selected treatment card was missing, then the next card was included in the sample. All data were recorded in real time in an electronic spreadsheet.

### LQAS analysis

We evaluated the impact of an LQAS system that classified each site either as *high* or as *low* data quality. The proposed system randomly sampled treatment cards and compared the sample of treatment cards to the register. Based on the results from these samples, sites were classified and only sites classified as low data quality would receive an exhaustive review of treatment cards and registers during supervision.

**Figure 1:**
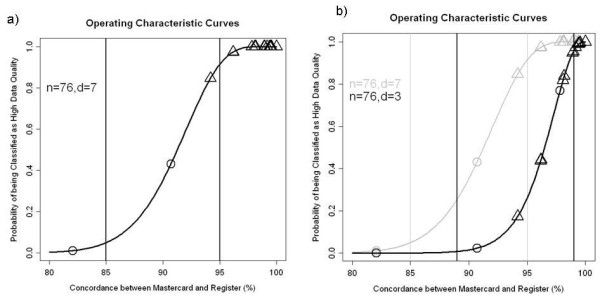
**Operating Characteristic Curves for the two LQAS classifications.** The curves plot the probability of site being classified as ‘high data quality’ based on the LQAS procedure for a given level of concordance between treatment cards and the register. Figure 1a is the plot for the 85%/95% classification. Figure 1b is the plot for the 89%/99% classification, superimposed on top of the 85%/95% classification. Each point represents a clinic, plotted at their true level of concordance between the treatment cards and register based on exhaustive review at the site. Circles indicate sites that are classified as ‘low data quality’ based on the LQAS classification. Triangles indicate sites that are classified as ‘high data quality’.

The sample size required was determined by LQAS methodology [20,21]. Thresholds for classification as determined by the M&E team at the Department for HIV and AIDS at the MoH, were: high data quality if there was 95% or better concordance between the treatment cards and register (with an allowable 10% risk of misclassification) and low data quality if concordance between the treatment card and register was 85% or less (with an allowable 5% risk of misclassification). Concordance between treatment card and register is defined as complete agreement on primary outcomes (current status and treatment regimen, if alive). Based on the binomial distribution, the minimum sample size required to meet these constraints was 76 (n = 76), with a corresponding decision rule of 7: i.e. a site with less than 7 discrepancies in the 76 sampled treatment cards would be classified as high data quality and a site with 7 or more discrepancies classified as low data quality (see Figure 1a). We also investigated a more conservative rule for site classification: with “high data quality” defined as 99% or more concordance between the treatment card and register and “low data quality” defined as 89% or less concordance. Fixing the sample size at 76, based on the binomial distribution we arrived at a decision rule of 3 for this sample size (see Figure 1b).

To evaluate the LQAS tool, random sampling of 76 records was done in parallel to the standard supervision process, where complete review and comparisons of treatment cards to registers was performed by the ART supervisors.

### Analysis

We compared basic site characteristics (patient burden, public versus private, length of time providing treatment, certificates of excellence) using frequencies. We also used counts to compare updated versus unchanged patient outcomes and treatment regimens, if alive. Secondary data outcomes (pill count and adherence) were restricted to patients alive and on treatment. These outcomes are reported with binomial confidence intervals at the site level, and aggregated using stratified sampling analysis, weighting by site size. Analyses and graphs were produced in Stata v.11 and R v.2.0.1.

### Ethical approval

All data were collected as part of standard clinical supervision and monitoring practices. Data were extracted from patient cards and registers and there was no direct contact with patients. This activity was an evaluation and quality improvement activity for the Department for HIV and AIDS. The whole process was reviewed and cleared by the Harvard School of Public Health and the Harvard Medical School IRBs.

## Results

Table 1 summarizes characteristics of the 19 sites included in the study. Four (21%) had initiated fewer than 100 patients at the site, six (32%) 101–500 patients, three (16%) 501–1000 patients, and six (32%) more than 1000 patients, a similar distribution to 249 out of 279 static ART clinics in the national treatment program that had initiated patients by the end of Q4 2009. The sample included fewer public clinics, and on average, the sampled sites had provided treatment longer than those in the general national treatment program. Only five (33%) of the sites received a certificate for excellent performance (with “performance” including measures of data quality and record keeping) at the previous supervision visit, a lower rate than in the national program.

**Table 1:** Site Characteristics

**Characteristics of sites**		**Sites included**		**National ART program**	
		**n = 19**		**n = 249**	
**Patient burden**					
	*100 patients or fewer*	4	21%	52	21%
	*101 - 500 patients*	6	32%	72	29%
	*501 - 1000 patients*	3	16%	39	16%
	*more than 1000 patients*	6	32%	86	35%
**Public clinics**		13	68%	199	80%
**Length of time providing treatment**^**†**^					
	*3 months or less*	3	16%	13	5%
	*3 months to 2 years*	3	16%	73	29%
	*2 to 4 years*	3	16%	103	41%
	*more than 4 years*	10	53%	60	24%
**Certificate given at last supervision visit**		(n = 15)		(n = 204)	
		5	33%	98	48%

^**†**^ Restricted to public sector sites. Originally, private and public sector sites were supervised separately. As a result, the national ART program records do not document length of time providing treatment for all private sector sites and these were excluded.

A total of 16,561 individuals had ever registered for ART at all sites. The exhaustive comparisons and cleaning of the treatment cards to the register took 28.5 hours total for the 19 sites. Extrapolating to all sites, we estimate 374 team-hours are dedicated to data cleaning at the 249 sites every quarter. During the exhaustive review, the supervision team identified few discrepancies in outcomes and treatment regimens when comparing the treatment card and register (median percent of records with discrepancies, 1.0%; IQR: 0.5%-3.0%). Of the 210 discrepancies between records (1.2%) in outcomes, a majority (58%) were incorrect in both the treatment card and the register, often as a result of a patient who had defaulted treatment and neither record had been updated. For 34% of the outcome errors, the treatment card was correct (with the register not updated), and in the remaining 8% the register was correct.

For the 11,034 patients alive and on treatment at the sampled sites, only 50 (0.4%) had discrepancies in treatment regimens recorded in their treatment card and register. Most often (94%), regimen changes had been recorded in the treatment card, but not updated in the register; the remaining were correct in the register (4%) or neither the treatment card nor the register (2%). For the 2,605 patients who initiated treatment in Q4 2009, 378 (15%) had discrepancies in their registration data (gender, age group, transfer status, TB history). The treatment card was determined correct in 71% of these errors, the register in 21% and neither in 8%. Usually this resulted from incorrect WHO clinical staging determined from the documented clinical conditions (where a person with a Stage IV condition would be listed as Stage III or vice versa), or TB status was incorrectly recorded as current instead of TB in the previous two years.

Since summary measures on outcomes and treatment regimens are extracted from the register, we explored the impact of relying on the registers in their original state versus the registers after a full review and update. Using the register without updates led to a 1.2% overcount in the number alive and on treatment, and a 5.6% undercount in the number of defaulters (Table 2). The uncorrected register data also overcounted by 0.4% the number on first-line treatment, and undercounted those on a zidovudine substitute and efavirenz substitute regimens by 3.6% and 9.7%, respectively. None of the site factors identified previously [19] were significant predictors of low data quality, possibly due to low power since so few sites were visited (results not shown).

**Table 2:** Impact of LQAS classification systems

	**System without any review of data quality**	**LQAS Triaging**	**LQAS Triaging**
		**Upper threshold = 95%**	**Upper threshold = 99%**
		**Lower threshold = 85%**	**Lower threshold = 89%**
n (sample size)	N/A	76	76
d (decision rule)	N/A	7	3
Number of sites above the upper threshold	N/A	16	9
*Number classified as high data quality*	N/A	16	9
Number of sites below the lower threshold	N/A	1	1
*Number classified as low data quality*	N/A	1	1
Differences between updated and non-updated data			
Alive	+1.2%	+1.2%	+0.7%
Default	−5.6%	−5.8%	−2.8%
1st Line Rx	+0.4%	+0.3%	+0.1%
Substitute, AZT	−3.6%	−2.8%	−1.5%
Substitute, EFV	−9.7%	−9.7%	−3.1%
Time Required for Review	N/A	8 hours;5 minutes	13 hours;19 minutes

Design and error rates in reporting under systems with a) no review process, b) LQAS classification system with p_u_ = 0.95 and p_l_ = 0.85, and c) LQAS classification system with p_u_ = 0.99 and p_l_ = 0.89. AZT = zidovudine. EFV = efavirenz.

At 16 sites, we randomly sampled 76 patient treatment cards. The remaining three had fewer than 76 cards inspected because fewer than 76 patients had ever initiated treatment at that site. At these three sites, the classification was made on the exhaustive review. LQAS classification of sites with the 85%/95% thresholds would have reduced the time needed for record review from 28.5 to 8 hours (see Table 2). Based on the exhaustive review, 16 sites had “high data quality” (95% or more agreement between treatment cards and register entries) and all of these sites would have been correctly classified using the LQAS system (Table 2 and Figure 1). “One site had less than 85% concordance between records, and this site would have been classified as low data quality via LQAS. For the two sites with concordance between 85-95%, one site would have been classified as low data quality. Because only two sites were classified as “low data quality” and thus would have received exhaustive review, there were very few updates to the aggregated data extracted from the register. The only marked improvement was a reduced undercount of numbers on the substituted zidovudine regimen (from 3.6% to 2.8%).

We also investigated the more conservative rules for site classification, with “high data quality” defined as 99% or more concordance between the treatment card and register and “low data quality” defined as 89% or less concordance. The nine sites with greater than 99% concordance based on the exhaustive inspection were correctly classified as “high data quality” by LQAS. The one site with “low data quality” was also correctly classified. The subsequent exhaustive review at the one additional site identified with this more conservative classification rule improved the accuracy of the aggregate measures extracted from the register, halving the percent errors on nearly all measures and reducing the undercount of efavirenz substitutes to only one third of its previous size (from 9.7% undercount to only 3.1% undercount). This increased accuracy required half of the time currently required for chart review at sites, though it increased the time by 50% over the less conservative (85%/95%) LQAS approach.

Table 3 reports the site specific estimates of side effects and adherence in patients alive and on treatment for the 16 sites where sampling was employed (sites with fewer than 76 patients were excluded). At these 16 sites, 41% to 97% of the sampled treatment cards were from patients who were alive and on treatment. Of those alive, between 62-100% had presence or absence of a side effect recorded. For those with recorded side effects, percent with side effects range from 0-27%, with a median of 3.3%. Based on the sample, the estimated proportion of patients alive with side effects was 7.2% (95% CI: 4.8-9.6%). For adherence, pill counts are only conducted on individuals alive and on 1^st^ line treatment, 39-92% of the original sample. For each site, 60-100% of eligible individuals had pill counts recorded and amongst these individuals, patient adherence ranges from 68-100%, with a median of 85.3%. The estimated proportion of patients alive that were adherent was 86.3% (95% CI: 82.8-89.8%).

**Table 3:** Secondary analysis of side effects and pill counts

		**Side effects**				**Adherence**				
	**Number alive in sample**	**Number with side effects recorded**	**Percent with adverse side effects**	**95% Confidence interval**		**Number alive and on 1st line in sample**	**Number with PC recorded**	**Percent with good adherence**	**95% Confidence interval**	
Site 1	49	47	2.1%	0.1%	11.3%	49	46	69.6%	54.2%	82.3%
Site 2	42	26	7.7%	0.9%	25.1%	39	29	89.7%	72.6%	97.8%
Site 3	47	41	14.6%	5.6%	29.2%	43	39	94.9%	82.7%	99.4%
Site 4	59	55	1.8%	0.0%	9.7%	57	47	87.2%	74.3%	95.2%
Site 5	58	55	0.0%	0.0%	6.5%	56	53	77.4%	63.8%	87.7%
Site 6	45	36	5.6%	0.7%	18.7%	39	31	80.6%	62.5%	92.5%
Site 7	44	44	0.0%	0.0%	8.0%	44	43	100.0%	91.8%	100.0%
Site 8	32	28	7.1%	0.9%	23.5%	30	28	85.7%	67.3%	96.0%
Site 9	44	33	15.2%	5.1%	31.9%	32	13	92.3%	64.0%	99.8%
Site 10	39	38	2.6%	0.1%	13.8%	39	39	74.4%	57.9%	87.0%
Site 11	31	27	3.7%	0.1%	19.0%	30	26	92.3%	74.9%	99.1%
Site 12	73	69	2.9%	0.4%	10.1%	70	65	67.7%	54.9%	78.8%
Site 13	41	34	2.9%	0.1%	15.3%	38	33	84.8%	68.1%	94.9%
Site 14	74	59	0.0%	0.0%	6.1%	65	56	85.7%	73.8%	93.6%
Site 15	57	49	26.5%	14.9%	41.1%	39	32	78.1%	60.0%	90.7%
Site 16	48	45	6.7%	1.4%	18.3%	41	34	76.5%	58.8%	89.3%
**Total**			**7.2%**	**4.8%**	**9.6%**			**86.3%**	**82.8%**	**89.8%**

## Discussion

Because of the extensive use of the national ART program data for management and analysis of patient care, ensuring the quality of this data is critical. Poor data can lead to imprecise budgeting and procurement of drugs, incorrect inference on program performance, and inappropriate adaptations to program implementation and, ultimately, poor patient care. Like other countries, Malawi has strived to improve and maintain data quality. As evident in these results, the overall level of quality is currently high for critical elements, but the system in place to produce this quality is expensive, time consuming, and can be redundant as many sites have already reviewed and cleaned data prior to supervision visits. Excluding travel time, nearly 750 team-hours every three months are spent at sites, and this time burden will only increase as more patients initiate treatment. We attribute half of this time solely to the data cleaning process and reducing the time for data cleaning can translate into budget savings for the ART program or, perhaps more importantly, into reallocation of time during supervision visits to more crucial activities such as mentoring or discussing difficult cases. With the LQAS system, we save time while maintaining the current standards for data quality. Other research highlights the challenges and associated consequences of poor data quality in health programs and each provides a method for measuring data quality, including some that rely on LQAS [19,22-34]. However, one unique benefit of this LQAS-based system is that the assessment is integrated into routine supervision. The simplicity of the procedure allows the classification to happen on site and immediately links to actions that improve data quality, such as the exhaustive data review in this case.

Based on these results, we recommend the following data cleaning strategy for the ART program in Malawi.

1. *Complete an exhaustive data cleaning process at sites with fewer than 500 patients ever initiated.* These sites should have an exhaustive review because error rates are often higher at smaller, less experienced sites such as these, because they have less practice with forms and are unlikely to have a designated data clerk. Further, the review at small sites is less time consuming, reducing the benefit of a sampling process.

2. *Exhaustive review of all patients initiating in the last quarter at every site.* Basic patient characteristics are collected once at registration and had high error rates (15%). Correcting these errors at the first supervision visit after initiation prevents them from recurring across future reports.

3. *For the public sites with more than 500 patients, sample 76 treatment cards from patients who initiated treatment before the last quarter and compare these against the register. Only complete an exhaustive review if 7 or more inconsistencies are identified.* The LQAS system with 85%/95% thresholds maintains data quality sufficient for program purposes and minimizes time required for data review/cleaning. We also recommend collecting information on secondary outcomes such as pill count and side effects during the sampling process.

Because time required for the exhaustive review was not separated by new and formerly registered patients, we could not estimate the exact time savings of this system. However, we expect the overall savings to remain significant since less than 16% of records were from new initiates. Further, the estimated time savings attributable to LQAS reviews at the small sites was small relative to the overall time savings (3 of the 20.5 hour reduction). The extrapolation regarding number of team hours taken to clean data each quarter assumed the same average quality of data across all clinics in Malawi, which given the purposive nature of our sampling may not be the case. Another limitation of this study, and perhaps the LQAS system for supervision, is the skipping of the patient treatment cards if the card was not available during the sampling procedure. Out of the records sampled, an average of 1.4 records per site were missing and skipped at the time of the visit. While some of these records were simply lost, often the records were not available because the patient was in the clinic receiving care during the supervision visit. Since most discrepancies were for patients who are no longer alive and on treatment, or who have defaulted, excluding these records likely increases the chance of classifying sites as having poor data quality and following up with a exhaustive data review. An additional limitation of this study (and the proposed supervision system) is the missing data on secondary outcomes from patient records. This likely biases the estimates of secondary outcomes, especially side effects where no record of side effects often suggests none are detected. We believe that active recording of secondary outcomes through the proposed supervision system could actually lead to improved documentation, as clerks and health workers will receive feedback during the process if the data is not recorded. Finally, while we believe the effect to be minimal since sites were not informed of the study team ahead of time, there could be some Hawthorne effect on the rigor of the supervision process. The Hawthorn effect could also affect the speed and accuracy of the ART supervision team, which would impact our estimates of time required for data cleaning as well as time saved using the LQAS system.

Additional concerns are raised when proposing a sampling approach for monitoring data quality. One is that failure to identify low data quality sites translates into inaccurate aggregated reports. The proposed algorithm has been designed to minimize this risk – sites with 85% or lower concordance between the treatment card and register have less than a 5% risk of being classified as high data quality at a single visit. As the data quality gets worse the chance of misclassification becomes even smaller, decreasing to less than 1% if the concordance goes down to 80%. This thus results in a very small potential for bias in the aggregated measures. Another concern is that sites may accumulate errors over time or that the site clerks may become less diligent if the exhaustive review is not completed. However, because the LQAS process is repeated independently each quarter, sites with degrading quality, if not captured at one visit, will likely be captured at future visits. Further, during previous rounds of supervision, clerks and health workers have expressed dissatisfaction at the automatic exhaustive review noting the duplication of their efforts and the impression that the supervision teams do not trust their work. The LQAS system addresses these concerns by prioritizing and conducting exhaustive reviews only at sites with poor data.

## Conclusion

In this paper, we describe the development of a simple LQAS system to assess data quality of sites. The LQAS classification is conducted independently at each visit; however the method could be extended so that performance at previous data quality assessments can be incorporated into the decision to conduct exhaustive review at a subsequent visit. Further, the Department for HIV and AIDS may still opt for exhaustive review at sites under special circumstances, such as immediately following guideline changes or changes in personnel at a site. Before any LQAS-based system can be implemented, forms to support the LQAS process must be developed and supervision teams need to be trained. The LQAS system, although straightforward to implement, is more complicated than the current methods that are standardized across all sites. However, supervisors would most likely welcome the system due to the substantial time saved. The LQAS system is unlikely to be a long-term solution for rapidly expanding ART programs such as in Malawi, it would provide a mid-term solution to ensure adequate quality of data until a reliable and sustainable system, such as an electronic record system, could be introduced nationwide [35]. Further, the system illustrated here can be readily adapted by other national programs using paper-based systems to ensure high quality data.

## Abbreviations

ART, Antiretroviral therapy; LQAS, Lot quality assurance sampling; MoH, Ministry of Health; M&E, Monitoring and evaluation; PEPFAR, President's Emergency Plan for AIDS Relief; Q4, Quarter 4.

## Competing interests

The authors declare that they have no competing interests.

## Authors’ contributions

BHG, LT, SM, MP, and AJ participated in the study design, planning, data collection, interpretation, and writing of the paper. BHG and SM led the analysis. FMC, SM, ZC, and EJS supported the planning, interpretation of results and editing of the paper. All authors read and approved the final manuscript.

## Pre-publication history

The pre-publication history for this paper can be accessed here:

http://www.biomedcentral.com/1472-6963/12/196/prepub
